# Diversity and Communities of Foliar Endophytic Fungi from Different Agroecosystems of *Coffea arabica* L. in Two Regions of Veracruz, Mexico

**DOI:** 10.1371/journal.pone.0098454

**Published:** 2014-06-02

**Authors:** Aurora Saucedo-García, Ana Luisa Anaya, Francisco J. Espinosa-García, María C. González

**Affiliations:** 1 Posgrado en Ciencias Biológicas, Instituto de Ecología, Universidad Nacional Autónoma de México, Distrito Federal, México; 2 Departamento de Ecología Funcional, Instituto de Ecología, Universidad Nacional Autónoma de México, Distrito Federal, México; 3 Laboratorio de Ecología Química, Centro de Investigaciones en Ecosistemas, Universidad Nacional Autónoma de México, Morelia, Michoacán, México; 4 Departamento de Botánica, Instituto de Biología, Universidad Nacional Autónoma de México, Distrito Federal, México; UC Irvine, United States of America

## Abstract

Over the past 20 years, the biodiversity associated with shaded coffee plantations and the role of diverse agroforestry types in biodiversity conservation and environmental services have been topics of debate. Endophytic fungi, which are microorganisms that inhabit plant tissues in an asymptomatic manner, form a part of the biodiversity associated with coffee plants. Studies on the endophytic fungi communities of cultivable host plants have shown variability among farming regions; however, the variability in fungal endophytic communities of coffee plants among different coffee agroforestry systems is still poorly understood. As such, we analyzed the diversity and communities of foliar endophytic fungi inhabiting *Coffea arabica* plants growing in the rustic plantations and simple polycultures of two regions in the center of Veracruz, Mexico. The endophytic fungi isolates were identified by their morphological traits, and the majority of identified species correspond to species of fungi previously reported as endophytes of coffee leaves. We analyzed and compared the colonization rates, diversity, and communities of endophytes found in the different agroforestry systems and in the different regions. Although the endophytic diversity was not fully recovered, we found differences in the abundance and diversity of endophytes among the coffee regions and differences in richness between the two different agroforestry systems of each region. No consistent pattern of community similarity was found between the coffee agroforestry systems, but we found that rustic plantations shared the highest number of morphospecies. The results suggest that endophyte abundance, richness, diversity, and communities may be influenced predominantly by coffee region, and to a lesser extent, by the agroforestry system. Our results contribute to the knowledge of the relationships between agroforestry systems and biodiversity conservation and provide information regarding some endophytic fungi and their communities as potential management tools against coffee plant pests and pathogens.

## Introduction

Commercial coffee production relies mainly on the plant species *Coffea arabica* L. [1], a species native to the highlands of Ethiopia and Sudan [2]. In Mexico, this plant was introduced at the end of the eighteenth century and was incorporated into the local agrosystems, where coffee plants were cultivated under shade in diverse polycultures [3], [4]. At the end of the 1970s, the Instituto Mexicano del Café promoted the transformation of traditional coffee polycultures into technified plantations [3], and at least five coffee production systems have been recognized in Mexico according to their vegetation structures, floristic composition, and management level [3], [5]. Four of these five coffee agrosystems are shaded plantations: rustic or traditional, diverse polyculture, simple polyculture, and shaded monoculture. The fifth agrosystem is unshaded coffee monoculture [5]. These systems represent a gradient from the most traditional agroforestry system, with reduced management and a high proportion of native tree canopy, to a lower proportion of native trees and a higher percentage of commercial shade trees. Unshaded coffee monoculture is at the end of the gradient of intensive management [3], [5], [6].

The relationship between coffee agroforestry types and their biodiversity has been attracting attention over the past two decades [6], [7]. In general, studies on this topic have shown that shaded coffee plantations contain a higher level of associated biodiversity than unshaded coffee plantations [7]–[10]. In particular, it has been proposed that traditional shaded coffee systems can act as refuges for many species in regions where deforestation has drastically affected the original forests. This function could be decisive in biogeographically important areas where habitats have been severely transformed [3], [6]. From a landscape perspective, the shaded plantations and their associated biodiversity preserve regional ecological processes and provide important ecosystem services [3], [11], as opposed to the highly intensified unshaded plantations with reduced biodiversity. However, the conservation value of the different shaded coffee systems varies widely, depending on the taxonomic group of organisms that constitutes a part of the biotic community of coffee plantations [8], [12].

Studies on the fungal diversity associated with Mexican coffee plantations, especially arbuscular mycorrhizal fungi (AMF) [13], [14] and saprotrophic fungi [13], have reported no significant differences in fungal richness or fungal communities among different coffee plantations with a gradient of management intensity.

Other studies on the foliar endophytic fungi (EF) associated with coffee plants have been conducted in abandoned coffee plantations in Puerto Rico [15]; in various locations in Colombia, Hawai'i, Mexico, and Puerto Rico [16]; and in plantations in the center of Veracruz [17]. However, no studies have been conducted to investigate the effects of the different coffee agroforestry systems on the diversity of EF found on coffee leaves.

Endophytic fungi are microorganisms that live in plant tissues without causing apparent harm to the host [18], [19]; therefore, plants with EF are asymptomatic [20]. These fungi have been found inhabiting healthy tissues in all plants in natural ecosystems [21]–[23], and they represent, individually and collectively, a continuum of variable associations with their host plants, from mutualism to latent pathogenicity [20]. In woody plants, such as *Coffea arabica*, the fungal endophytes are transmitted horizontally [23]; the growth of these fungi is highly localized within particular plant tissues [24], [25]. With time, the EF accumulate in plant tissues, producing a heterogeneous mosaic of different species of endophytes in every plant organ [26], [27].

The EF communities (EFC) may be influenced by the traits of a given host plant [26]–[31], distribution of the host plant [22], [32], [33], and characteristics of the locality in which the plant grows [27], [34]–[36]. Studies on the EFC of cultivable host plants have shown variability among these communities based on the regions in which the plants are cultivated [37], [38]; however, the variability in EFC among management systems is still poorly understood.

The aim of the present study was to analyze whether the coffee agroforestry system and the region where the coffee is cultivated could influence EF diversity and the EFC associated with *Coffea arabica* leaves. We analyzed and compared the colonization rates (CR), richness, diversity, and fungal communities of the EF inhabiting the leaves of *Coffea arabica* in different agroforestry systems in two different coffee regions, Huatusco and Coatepec, in the center of the state of Veracruz, Mexico. We selected coffee plants from a rustic plantation and from a simple polyculture in each of the coffee regions. We isolated 471 foliar endophytes, which we assigned to 31 morphospecies, from the four selected coffee plantations. We found differences in CR and EF diversity between the coffee regions, as well as differences in richness between the two different agroforestry systems of each region. The analysis of EFC similarity revealed that the endophytic communities in the two coffee plantations in Coatepec did not vary, while the EFC of the coffee plantations in Huatusco were different. There were similarities in the EFC of the rustic plantations and in the EFC of the simple plantations.

Studying the fungi associated with the phyllosphere of coffee plants enable us to recognize the presence of coffee pathogens and to evaluate, in future studies, the potentiality of some EF and EFC in controlling pathogens or pests of this important cultivated plant.

## Materials and Methods

### Ethics Statement

No specific permits were required for the described field studies. This work did not involve endangered or protected species.

### Study sites

**Figure 1:**
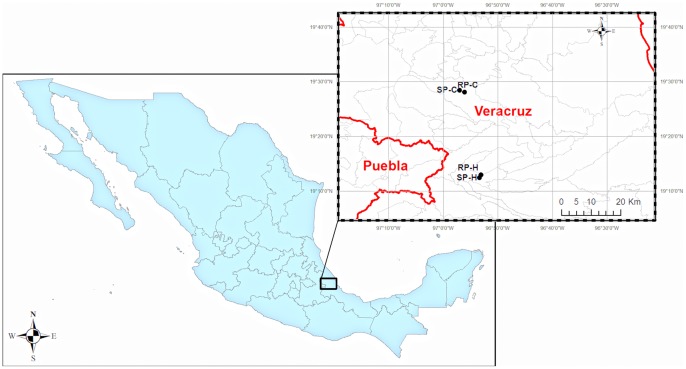
Map of survey area: geographical locations of the four coffee plantations, Veracruz, Mexico. RP-C: rustic plantation Coatepec, SP-C: simple polyculture Coatepec. RP-H: rustic plantation Huatusco, SP-H: simple polyculture Huatusco.

Four shaded coffee plantations from the central coffee-growing region of Veracruz, Mexico were selected as field study sites (Fig. 1). Two plantations were located in the Coatepec region and two were located in the Huatusco region. Although both of these regions are located in the tropical montane cloud forest, there are some differences between them. Both regions have a humid climate (A) C according to the classification of Köppen modified by García (1998) [39], but Coatepec experiences rainfall all year round (A) C (fm), whereas precipitation is seasonal in Huatusco (A) C (m). Another difference between the two regions is that the Coatepec region is located in the montane cloud forest, whereas the Huatusco region is located at the border of the montane cloud forest and the tropical deciduous forest [40]. In each region (Fig. 1), we selected one rustic plantation and one simple polyculture plantation according to a classification based on the vegetation structure of each plantation [5], [41].

In rustic plantations, coffee is grown under the shade of native vegetation, such as *Heliocarpus* sp., *Quercus sartorii, Myrsine coriacea*, and *Trema micrantha*, with some selected shade trees, such as *Inga* spp. and *Citrus* spp. The canopy has a high degree of vertical stratification, and the vegetation contains abundant epiphytes [3], [5], [41]. These rustic plantations have low management intensity; the farmers predominantly use alternative management techniques, such as manual weed control and occasional pruning of coffee plants, with no addition of fertilizer.

In simple polyculture coffee plantations, the canopy of the native trees of the forest are removed and replaced with selected shade trees, such as legumes (*Inga* spp.), and crop species with commercial value, such as *Citrus* spp. and *Musa* sp. [5], [41]. In this management system, the farmers apply agrochemicals, such as fertilizers and pesticides, to the coffee plants.

### Sample collection and endophyte isolation

In November 2009, ten coffee plants, spaced at least 2 m apart, were chosen from each of the four plantations. To homogenize the sample collection, healthy, mature leaves from north-facing branches growing in the middle of the selected coffee plants were collected. Four coffee leaves from the middle part of the selected branches were collected, stored in a cool box above ice, and processed within 48 h.

Isolation of the EF was performed using the surface sterilization method for coffee leaves [15], washing them in running water and surface sterilizing them with sequential solutions of 70% ethanol (1 min), 2.6% sodium hypochlorite (3 min), and 70% ethanol (1 min). The leaves were then rinsed with sterilized, distilled water. Two round fragments (2 mm in diameter) were cut from the base, middle, and apex of the lamina of each leaf with a sterile leaf punch. Each surface-sterilized fragment was placed separately in a Petri dish with oatmeal agar (OA: 30 g oatmeal, 20 g agar, and 1 L distilled water) supplemented with chloramphenicol (50 mg/L). The leaf fragments were pressed briefly into the surface agar in the margins of the Petri dishes to create leaf prints and determine whether the superficial sterilization was successful. The Petri dishes were incubated at room temperature under natural light. The growth of the fungi and the leaf imprints were checked every three days for one month. The tips of hyphae from different fungi emerging from the same leaf fragment were subcultured on OA plates.

### Morphological identification of endophytic fungi

All fungi isolates were examined after five and ten days and were grouped into morphotypes based on the following morphological traits: shape of the mycelium, texture of the mycelium surface, color of the fungi, production of pigments and their diffusion into the medium, production of spores, and mycelium growth rates in the OA plates.

To enable morphological identification, 1 to 15 isolates were selected from each morphotype, depending on abundance of each morphotype. Selected isolates were cultured on OA and potato dextrose agar (PDA: 200 g scrubbed and diced potatoes, 15 g dextrose, 20 g agar, and 1 L distilled water) plates. The EF that did not sporulate on these media were transferred to PDA, OA, and malt extract agar (MEA 2%) plates and to plates with leaf extracts of healthy, mature coffee leaves (10% wt/vol) to activate sporulation.

We examined the above-mentioned traits of the mycelium morphology of the fungi isolates and their microscopic characteristics. The characteristics evaluated for the anamorph type were conidiomata, conidiogenous cells, conidiophores, and conidia morphology (e.g., size, color, shape, ornamentation), and the characteristics evaluated for the teleomorph type were sporomata, their associated structures, and spore morphology. The strains of isolated and identified endophytic fungi are part of a fungal collection of the Laboratorio de Alelopatía, Departamento de Ecología Funcional, Instituto de Ecología, Universidad Nacional Autónoma de México; the strains are freely available.

Following the method described in Waller et al. (1993) [42], the fungi belonging to the *Colletotrichum* genus were cultured in basal media supplemented with a different carbon source (i.e., ammonium tartrate or citric acid). Using that media enabled us to evaluate the substrate utilization of the isolated *Colletotrichum* strains and differentiate among species, as some species, such as the coffee pathogen *Colletotrichum kahawae*, cannot metabolize tartrate and citric acid [42], [43].

The fungi were classified into morphospecies based on their growth rates and morphological and microscopic characteristics. Isolates that did not sporulate were identified as morphotypes (Mycelia sterilia), based on their morphological characteristics. The relative frequency of EF for each coffee plantation was calculated as the abundance of a given species divided by the total number of fungi isolated from each coffee plantation.

### Colonization and isolation rates

Colonization rate (CR) was calculated as the number of fragments from which one or more EF was isolated, divided by the total number of incubated fragments [44]. The isolation rate (IR) was defined as the number of EF isolated, divided by the total number of fragments incubated [45], [46].

CR and IR were analyzed using a two-way ANOVA test, with regions (Huatusco and Coatepec) and agroforestry category (rustic and simple polycultures) as the factors. The difference between mean values was evaluated using Tukey's honestly significant differences (HSD) test. The statistical analysis was performed using Statistica software 8.0.

### Diversity analysis

Because leaves serve as largely discrete, relatively uniform, bounded habitats to the microbes that inhabit them [47], we considered each leaf as a sampling unit, and the EF isolated from each leaf was considered to represent an EFC. To assess species richness and evaluate the sampling intensity, the observed and Jackknife 1 expected richness of EF for each coffee plantation were calculated with EstimateS software [48], using 1000 runs of bootstrapping with replacement. Jackknife 1 has proven to be a reliable estimator for various organisms [49], including fungi [35], [50]. The observed and expected species accumulation curves were plotted using individual leaves as the unit for each plantation.

The richness and distribution of EF found in each coffee plantation were examined using the range diversity (RD) analysis developed by Arita et al. (2012) [51]. Using this method enabled us to analyze the richness and distribution of EF found in the coffee leaves of each plantation, as well as to evaluate the association and co-occurrence of morphospecies. The RD analysis was performed following the R script of Arita et al. (2012) [51], using the R program [52]. Analysis of variance was used to test for differences in endophyte richness between coffee plantations and coffee regions.

Following the R script of RD analysis [51], we computed the variance–covariance matrices among morphospecies and among leaves from each coffee plantation. Using the matrices enabled us to analyze the co-occurrence patterns of morphospecies in the coffee leaves from each plantation.

To test a possible association among species and a possible clustering of leaves in terms of shared species in each coffee plantation, we computed the ratio of variance for species (V_sp_) and for leaves (V_lv_) according to the method of Arita et al. (2012) [51]. If the ratio–variance value is higher than 1, there is a positive association among morphospecies or a greater similarity in morphospecies composition among leaves. If the ratio–variance value is less than 1, there is a negative association among morphospecies or no similarity in EFC among the leaves from each coffee plantation. We contrasted the values of ratio–variance obtained in the RD analysis (empirical values) with the values obtained with null models, which contrast real world assemblages against hypothetical patterns generated by randomizing some variables of a model [51]. The empirical values of V_sp_ were contrasted with null models in which we maintained the original frequency of EF richness found in every coffee plantation, but we assigned leaves to species randomly. The empirical values of V_lv_ were contrasted with null models in where we maintained the original frequency of number of species found in each coffee leaf, but we generate permutations to simulate the random assignment of species to leaves.

The Fisher's alpha and Shannon diversity (H') indexes of fungal endophyte species found in each coffee leaf were calculated with EstimateS software [48], using 1000 runs of bootstrapping with replacement to generate 95% confidence intervals for each diversity value. The statistical differences in foliar EF diversity between coffee plantations and regions were analyzed using an ANOVA test of two factors, and the differences between mean values were evaluated using Tukey's HSD test.

EFC similarities found among the coffee plants from the four plantations were analyzed using non-metric multidimensional scaling (NMDS) plots. The NMDS plots display the dissimilarities among EF communities graphically, and the distances between them on the plot represent their relative dissimilarity [53]. Three NMDS plots were constructed, each based on a different calculated ecological similarity index: Jaccard's index, based on the presence/absence of taxa among trees [54]; Bray–Curtis coefficient, based on the incidence and abundance of taxa found in the trees [54]; and Euclidean distance, a dissimilarity measure based in quantitative abundance data and the joint absences of taxa isolated among trees [54]. For each ecological similarity index calculated, a one-way analysis of similarity (ANOSIM) and a Bonferroni-corrected pair-wise comparison were performed to test for significant differences in the EFC of the coffee plants among plantations. The ANOSIM test uses a statistic (R) that ranges from 0 to 1. A zero R value represents a similarity between objects in different groups, and R values greater than zero indicate that objects are more dissimilar between groups than within groups [53]. The NMDS plots and ANOSIM analysis were performed using PAST software [55].

## Results

### Abundance and diversity of endophytic fungi

A total of 479 EF were isolated from the 80 *Coffea arabica* leaves collected. At least one EF was isolated in each the leaves examined, with one exception: no fungi were isolated from one coffee leaf from the simple polyculture plantation in Coatepec (SP-C). Therefore, that leaf was not included in the statistical analysis. The ANOVA test showed significant differences in CR and IR between the two regions (CR: F = 15.1249, p = 0.0002; IR: F = 14.523, p = 0.0003) and between the two agroforestry types (CR: F = 6.4181, p = 0.0134; IR: F = 8.3885, p = 0.0049). According to the post-hoc tests (Table 1), CR was significantly higher in Huatusco's rustic plantation (RP-H) and simple polyculture (SP-H) than in SP-C. In addition, significant differences in IR were found between the coffee leaves from RP-H and both Coatepec plantations, rustic (RP-C) and SP-C. There were no significant differences in CR or IR between the two agroforestry systems of each coffee region.

**Table 1:** Colonization rate (CR) and isolation rate (IR) of endophytic fungi in the four coffee plantations.

	Huatusco		Coatepec	
	Rustic plantation	Simple polyculture	Rustic plantation	Simple polyculture
CR	0.88±0.04**^a^**	0.80±0.05**^a^**	0.73±0.05**^ab^**	0.57±0.05**^b^**
IR	1.30±0.09**^a^**	1.01±0.07**^ab^**	0.93±0.07**^b^**	0.75±0.09**^b^**

Mean of CR ± standard error and mean of IR ± standard error of fungal endophytes isolated from the four coffee plantations. Different letters indicate significant difference between coffee plantations at the p<0.05 level.

The 479 isolated EF were assigned to 31 morphospecies, including both identified genera and unidentified types. The relative frequencies of isolation of these fungi are shown in Table 2, and their descriptions are shown in Table S1. The particularly common EF genera were *Colletotrichum* and *Xylaria*.

**Table 2:** Morphospecies of endophytic fungi from the four coffee plantations and their frequencies.

	Huatusco		Coatepec	
Taxon	Rustic	Simple polyculture	Rustic	Simple polyculture
*Alternaria citri*				0.02
*Beauveria brongniartii*	0.01			
*Colletotrichum* aff *brassicicola*	0.03	0.06	0.03	0.03
*Colletotrichum gloeosporioides* 1	0.21	0.32	0.36	0.33
*Colletotrichum gloeosporioides* 2	0.13	0.01	0.03	0.07
*Colletotrichum musae*	0.04	0.02	0.01	
*Colletotrichum* sp. 1	0.03	0.07	0.02	0.04
*Coniosporium* sp.	0.03	0.02	0.10	0.08
*Cryptopsoriopsis corticola*	0.03		0.02	0.00
*Cryptopsoriopsis* sp. 1	0.04		0.02	0.07
*Diplodia* sp.	0.03	0.12	0.09	0.07
*Glomerella cingulate*	0.15	0.12	0.04	0.07
*Guignardia mangiferae*	0.03	0.06	0.09	0.01
Hyphomicete 1		0.01		
Hyphomicete 2				0.02
Mycelia esterilia 1	0.01	0.02	0.04	0.03
Mycelia esterilia 2	0.03			
Mycelia esterilia 3	0.01			
Mycelia esterilia 3		0.01		
Mycelia esterilia 4			0.01	
Mycelia esterilia 5			0.01	
*Paecilomyces* sp.	0.03	0.02	0.01	0.03
*Phomopsis arnoldiae*	0.02			0.01
*Phomopsis* sp.		0.02		
*Xylaria* 1.	0.08	0.05	0.03	0.04
*Xylaria* 2.	0.06	0.07	0.08	0.07
*Xylaria* 3	0.02		0.01	
*Xylaria* 4.	0.01			
*Xylaria* 5.			0.02	
*Xylaria* 6		0.01		

The *Colletotrichum gloeosporioides* morphospecies was separated into two types (1 and 2) based on spore and conidiogenous cell size and mycelium traits. In the *Xylaria* genus, we recognized six morphotypes according to their morphological characteristics. Due to a lack of spore production, eight EF morphotypes, which represented only 5% of the isolated fungi, could not be identified; they were named Mycelia sterilia 1–8.

Interestingly, we observed changes in the appearance of the fresh leaf fragments cultured in the Petri plates according to the fungi isolated from them. The leaf segments in which the genus *Xylaria* was isolated withered after a few days of culture, while the leaf segments colonized by *Coniosporium* remained green in the Petri plates for almost one month.

**Figure 2:**
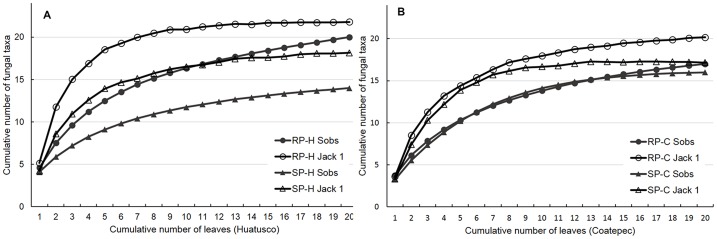
Rarefaction curves for fungal endophytes isolated from coffee leaves from the four coffee plantations. Species accumulation curves (gray circles and gray triangles) and Jackknife 1 estimated richness (open circles and open triangles) of rustic plantations (circles) and simple polycultures (triangles) of Huatusco region (A) and Coatepec (B). RP-H: rustic plantation Huatusco; RP-C: rustic plantation Coatepec; SP-H: simple polyculture Huatusco; SP-C: simple polyculture Coatepec.

Accumulation curves of observed species richness (gray triangles and circles) and Jackknife 1 estimated richness (open triangles and circles) are shown in Figure 2. Observed and estimated richness were higher in RP-H (circles in Fig. 2A) than in SP-H (triangles in Fig. 2A). Estimated richness (open circles and triangles) was higher than observed richness (gray circles and triangles) in all four coffee plantations.

The only coffee plantation in which the rarefaction curve reached the saturation point was the SP-C (Fig. 2B). Richness estimator analysis of this plantation showed that the estimated morphospecies richness was similar to the observed richness, indicating sufficient sampling work in SP-C.

The rarefaction curves of the other three plantations did not reach the saturation point, suggesting that endophyte richness was not fully recovered. The Jackknife 1 expected richness for those locations was higher than the observed richness, indicating that species richness was not exhaustively sampled.

The results of the RD analysis summarized in Table 3 show the total EF richness isolated from each coffee plantation and the number of leaves from which at least one EF was isolated. Analysis of variance showed that EF richness was higher in the coffee leaves from the Huatusco region than in the coffee leaves from Coatepec (F = 11.7512, p = 0.0009). In each region EF richness was higher in the rustic plantation than in the simple polyculture (F = 4.1639, p = 0.0448). There were coffee leaves from Coatepec from which only one EF morphospecies was isolated, and the maximum was six morphospecies per leaf. In contrast, the Huatusco coffee leaves had a minimum of two or three morphospecies per leaf, with a maximum of nine different morphospecies per leaf.

**Table 3:** Richness-distribution analysis of the endophytic fungi isolated from each coffee plantation.

	Huatusco		Coatepec	
	Rustic plantation	Simple polyculture	Rustic plantation	Simple polyculture
Total Richness	21	17	19	16
Leaves with endophytes (EF)	20	20	20	19
Mean of EF richness in coffee leaves	5.10±0.40	4.15±0.26	3.70±0.26	3.32±0.35
Min-Max of EF richness by leaf	3–9	2–6	1–5	1–6
Mean of distribution of EF	4.86±0.82	4.88±1.21	3.84±0.81	3.94±0.86
Min-Max distribution of EF	1–15 leaves	1–20 leaves	1–14 leaves	1–16 leaves
Ratio–variance for species (V_sp_)	0.949	0.621	0.641	1.311
Ratio–variance for leaves (V_lv_)	3.826	6.535	3.949	3.792

Values of total richness, media of richness ± standard error, distribution, media of distribution ± standard error, and ratio–variance of endophytic fungi in the four coffee plantations.

On average, each EF morphospecies was isolated in five leaves from the Huatusco plantations and in four leaves from the Coatepec plantations. The minimum number of leaves in which a given morphospecies was isolated was one leaf, and the maximum was 14–20 leaves. The most widespread EF species in all four coffee plantations was *Colletotrichum gloeosporioides* 1.

The V_sp_ value, which indicates the degree of association among different species [56], was 1 in RP-H and SP-C (Table 3). Those values were similar to the V_sp_ of null models with 10 iterations (RP-H: V_sp_ = 1.01±0.04; SP-C: V_sp_ = 1.02±0.28). Empirical and null models showed no association among morphospecies in those plantations. In RP-C and SP-H, the empirical V_sp_ values were less than 1, while in the simulations values V_sp_ were 1 (RP-C: V_sp_ = 1.00±0.16; SP-H: V_sp_ = 1.10±0.07). Those results indicate negative associations among EF species in RP-C and in SP-H. Negative covariances were observed between *Colletotrichum gloeosporioides* and *Xylaria*, *Xylaria* and *Coniosporium* sp., and *Colletotrichum gloeosporioides* and *Coniosporium* sp. In general, positive covariances were found among fungi isolated in low frequencies, such as *Paecilomyces* sp., which was only isolated from leaves in the presence of one Coelomycete endophyte. The V_lv_ value, which indicates EFC similarity among leaves from the same coffee plantation, was higher than 1 in all four coffee plantations (Table 3). Those results contrast with the V_lv_ values obtained with the null models (RP-H: V_lv_ = 0.87±0.18; RP-C: V_lv_ = 0.95±0.02; SP-H: V_lv_ = 1.29±0.16; SP-C: V_lv_ = 0.93±0.07), and indicates considerable similarity in the shared species of the leaves of each coffee plantation. The highest EFC similarity was found in the coffee leaves from SP-H.

The ANOVA test of diversity indexes of EF isolated from the coffee leaves showed significant differences in diversity between agroforestry systems (Fisher's alpha: F = 40.4381, p<0.0001; Shannon's diversity: F = 22.8818, p<0.0001) and with the interaction between coffee region and agroforestry system (Fisher's alpha: F = 43.9388, p<0.0001; Shannon's diversity: F = 40.3883, p<0.0001). The Shannon's diversity was different between coffee regions (F = 19.7207, p<0.0005), while the Fisher's alpha was not different between them (F = 0.4303, p = 0.5140) due to the variability in Fisher's alpha diversity in Huatusco region. Fisher's alpha EF diversity (Table 4) was significantly higher in the RP-H leaves than in the coffee leaves of the other three plantations; SP-H leaves had the lowest Fisher's alpha diversity value. Shannon's index of EF diversity was significantly higher in RP-H than in RP-C, SP-H, and SP-C; there were no significant differences in Shannon's index of EF diversity among RP-C, SP-H, and SP-C.

**Table 4:** Fisher's alpha and Shannon's diversity index of endophytic fungi in the four coffee plantations.

	Huatusco		Coatepec	
	Rustic	Simple polyculture	Rustic	Simple polyculture
Fisher's alpha	6.53±0.15 ^a^	5.05±0.10 ^c^	5.70±0.08 ^b^	5.73±0.11 ^b^
Shannon	2.40±0.03^ a^	2.07±0.03^ b^	2.08±0.03^ b^	2.13±0.03^ b^

Mean ± standard error. Different letters indicate significant difference between coffee plantations at the p<0.05 level with ANOVA test of interaction between coffee region and agroforestry system.

### Analysis of EFC similarity among coffee plantations

**Figure 3:**
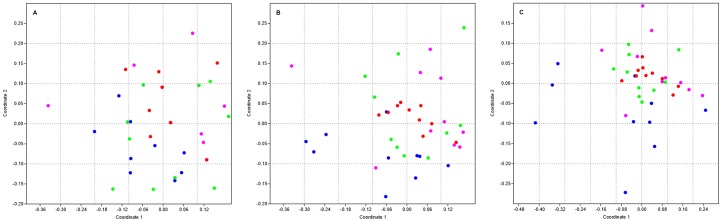
Non-metric multidimensional scaling (NMDS) plots of fungal endophytic communities from the four coffee plantations. NMDS plots based in Jaccard's index (A), Bray–Curtis coefficient (B), and Euclidean distance (C). Fungal endophytic communities of each coffee plantation are indicated by different colors: blue circles  =  rustic plantation Huatusco; pink circles  =  rustic plantation Coatepec; red circles  =  simple polyculture Huatusco; and green circles  =  simple polyculture Coatepec.

The NMDS plots (Fig. 3) with Jaccard's index had a stress (S) value of 0.34, the NMDS plots with the Bray–Curtis coefficient had a 0.24 S value, and the Euclidean distance NMDS plots had a 0.16 S value. In NMDS plots, S values higher than 0.3 indicate a poor configuration of dissimilarities among the EFC in a multidimensional space, while S values lower than 0.2 indicate a better configuration of the dissimilarities [53]. Regardless of the ecological similarity index analyzed, there was not a clear clustering of EFC isolated from the coffee plants among the different plantations.

The results of the similarity analysis summarized in Table 5 indicate a significant difference in the EFC of RP-H and SP-H analyzed with Jaccard's index (R = 0.356), the Bray–Curtis coefficient (R = 0.324), and Euclidean distance (R  =  0.258). Those results are better observed in the NMDS plots based on the Bray–Curtis coefficient (Fig. 3B) and Euclidean distance (Fig. 3C). Those figures show that the EFC of the different SP-H coffee plants are very similar (red circles in Fig. 3) and that those communities are different from the EFC isolated from the RP-H coffee plants (blue circles in Fig. 3).

**Table 5:** ANOSIM pairwise test of EFC isolated from the four coffee plantations.

Pairwise test	R (Jaccard's index)	R (Bray-Curtis coefficient)	R (Euclidean distance)
RPH - RPC	0.211	0.164	0.145
RPH - SPH	0.356*	0.324*	0.258*
RPH - SPC	0.181	0.124	0.186*
RPC - SPH	0.003	0.111	0.110
RPC - SPC	0.060	0.004	0.111
SPH - SPC	0.183	0.141	0.169

The ANOSIM test was based on three different ecological similarity indexes: Jaccard's index, Bray–Curtis coefficient, and Euclidean distance. RPH: rustic plantation Huatusco; RPC: rustic plantation Coatepec; SPH: simple polyculture Huatusco; SPC: simple polyculture Coatepec; R  =  rank similarities; *significant difference p>0.05.

The ANOSIM analyzed with the Euclidean distances also showed significant differences between the RP-H EFC and the SP-C EFC (R = 0.186). The NMDS plot based on Euclidean distance shows that the circles representing EFC from SP-C (green circles in Fig. 3C) are different to EFC from RP-H (blue circles in Fig. 3C).

The NMDS plots and ANOSIM based on the three ecological similarity indexes indicate that the EFC of the trees inhabiting the Coatepec region were similar, independent of the coffee agroforestry system. The similarity analysis also showed that the EFC isolated from each type of agroforestry system, rustic and simple polyculture, were similar, independent of the coffee region. There were similarities in the EFC of the rustic plantations (RP-H and RP-C) and in the EFC of the simple plantations (SP-H and SP-C).

## Discussion

In this study, we identified 31 morphotaxa from the 479 isolated endophytic fungi. The number of morphospecies found in our study is lower than the number found in the study by Santamaría and Bayman (2005) [15], who studied the EF of coffee leaves from Puerto Rico and collected the same number of coffee leaves as in the current study. This difference might be due to the number of sites sampled; we sampled four sites, while Santamaría and Bayman sampled six sites. Because the interpretation of EF richness is method-dependent [21], the difference in the number of sites could affect the number of morphospecies recovered. However, it is far more likely that the difference is due to the type of management practiced at the sites studied. We isolated EF from coffee leaves in producing plantations, while Santamaría and Bayman isolated foliar EF from coffee plants growing in a secondary forest and a botanical garden. The traits of the ecological environment of the site strongly influence EF diversity [57].

The species reported in the present study are ubiquitous taxa isolated mainly from plants inhabiting tropical regions [22], [25], [35], woody cultured plants [16], [58], [59], and plants growing outside their native distribution areas [32], [33]. The most common genera found in our study were *Colletotrichum* and *Xylaria*, which have often been isolated from coffee plants in American coffee-growing regions [15], [16]. Other EF genera reported in the present study have also been isolated from coffee plants growing in different countries; these genera include *Phomopsis* [60], *Beauveria* [61], *Alternaria* [62], *Coniosporium* [17], *Guignardia* [15], *Paecilomyces* [16], and the teleomorph of *Diplodia*, *Botryosphaeria* [16].

Knowledge of the diversity of EF in coffee leaves is very important in identifying phytopathogenic fungi that might live as endophytes during part of their life cycle [63], [64]. Of the EF isolated in the present study, the *Colletotrichum gloeosporioides* complex includes strongly aggressive pathogens, opportunistic pathogens [65], and endophytes [15], [16]. In Latin America, *Colletotrichum gloeosporioides* is associated with blister spot, or “mancha mantecosa,” in leaves [66]. The tartrate and citric acid biochemical tests conducted on isolates 1 and 2 of *Colletotrichum gloeosporioides* in this study, as well as the successful inoculation of these fungi in coffee plants without causing evident damage or disease (Velázquez-Bermudez, in preparation), show the endophytic nature of this species isolated from coffee leaves.

The genus *Xylaria* is recognized as a saprotrophic fungus [67], [68] and as an endophytic fungus of many plants [69], [70], included coffee plants [15], [16]. We observed that the coffee leaf segments in which the genus *Xylaria* was isolated withered in a few days of Petri dish cultures, and some of them produced stromata only over the dead leaf segment. This phenomenon might be evidence of the saprobic phase of the xylariaceous fungus [71]. According to Vega et al. (2010) [16], the endophytes of this genus might play a saprotrophic role in coffee plants after their senescence.

Some genera, such as *Beauveria*, which was only isolated from RP-H leaves, and *Paecilomyces*, are recognized as entomopathogenic fungi and have been isolated and tested on some coffee pests [72]. Further studies on the entomopathogenic role of these genera will be very important in elucidating their possible ecological relation in each agrosystem.

The analysis of EF abundance in the coffee plantations in the two regions studied indicated higher CR and IR values in the Huatusco plantations than in the Coatepec plantations. This finding indicates that the geographical region (with its respective vegetation, climate, and soil characteristics) exerts an important influence on the colonization and abundance of foliar EF in coffee plants. Other studies have also shown differences in the abundance of EF between different localities in agrosystems [38] and natural systems [30], [73].

On the other hand, we found no significant differences in CR or IR between the two agroforestry systems of each coffee region; however, we found higher CR and IR values in the rustic plantations than in the simple polycultures. Coffee plants in rustic plantations, as opposed to simple polycultures, are grown under a higher diversity of shade trees species, representing a more complex canopy. However, as shown in other studies, the canopy does not seem to influence the colonization and abundance of EF in plants growing in the understory [27], [74]. In agreement with our results, Arnold and Herre (2003) [27] found no differences in the CR of EF in plants growing under different canopy conditions (beneath the forest canopy and under cleared sites), although they found a higher number of fungal colony-forming units in the forest than in the cleared sites.

Besides the differences in canopy, the two coffee agroforestry systems also differ in their farming practices. However, the differences in management systems also do not seem to affect the colonization and abundance of EF. Similarly, Pancher et al. (2012) [37] found no differences in the number of EF isolated from grapevines (*Vitis vinifera*) cultivated in vineyards following different farming practices.

We found that the CR and IR of EF are not necessarily related to the diversity found in each sampling site. For example, Matsumura and Fukuda (2013) [75] reported higher frequencies of colonization of EF in trees inhabiting rural forests than in trees growing in a suburban forest in Japan. However, they did not find significant differences in Shannon's diversity index between these two forests. In contrast, we found similar colonization rates and abundance of EF in the Huatusco plantations, but the diversity values in the rustic plantation were higher than in the simple polyculture. On the other hand, we did not find significant differences in the EF diversity of the two Coatepec coffee plantations. Nevertheless, it is important to mention that the rarefaction curves for RP-C, RP-H, and SP-H did not reach saturation of morphospecies. This finding suggests that additional sampling efforts are needed in those plantations in order to obtain an accurate idea of the endophytic richness; the species that were not isolated might raise the diversity levels in those locations.

We found a higher total EF richness, a higher mean richness by leaf, and a higher expected richness in the rustic plantation than in the simple polyculture of each region. As mentioned previously, these results suggest that the complexity of the canopy and the alternative management techniques used in rustic plantations might influence the EF richness and diversity. Some studies have shown that the canopy might influence the richness and diversity of foliar EF growing in the understory [35], [76], [77]. A complex canopy could produce microclimatic conditions that promote a higher diversity of EF [76]. In rustic plantations, the complex canopy might also produce a higher diversity of litter types, and as those dead leaves appear to be a primary source of EF inoculum [36], [71], the diversity of litter types might also produce a higher diversity of EF propagules.

Various studies have been conducted on biodiversity conservation in different coffee production systems in the central region of Veracruz, where Huatusco and Coatepec are located [78]. Contrary to our findings, studies regarding the fungi associated with different coffee agroforestry systems, particularly arbuscular mycorrhizal fungi (AMF) and saprotrophic fungi, showed no differences in richness or diversity among the different agrosystems [13], [14]. It is possible that EF is more sensitive to agroforestry type than AMF and saprophytic fungi are.

In addition to the variability in richness and diversity of EF between coffee agrosystems, we found higher EF richness and diversity in RP-H than in the other three plantations, and a higher mean richness by leaf in the Huatusco plantations than in the Coatepec plantations. One reason for this finding is the difference in EF distribution in the two regions. Although we observed the typical distribution of EF reported in many studies on tropical and temperate forest trees [21], [35], [79], in which few EF morphospecies were frequent and the majority of morphospecies were found in low frequencies, we found some differences between regions. In general, the morphospecies in the Huatusco plantations had a broad distribution among the leaves, while the Coatepec morphospecies had a narrow distribution among the leaves. The differences in species distribution might influence the mean richness and diversity values of the coffee leaves. Furthermore, the Huatusco region (Fig. 1) is located at a lower longitude than Coatepec, and although it is immersed in a montane cloud forest, it runs adjacent to a tropical deciduous forest. These differences could influence the EF diversity of those plantations. Some studies have reported that Shannon's diversity index of EF increases when the longitude decreases [80]; this finding is consistent with our results for Huatusco and Coatepec. In the other hand, in Veracruz, there is also a higher diversity of other organisms, such as small- and medium-sized mammals [81] in the rustic Huatusco plantations than in the simple polycultures of Huatusco, and in the different agroforestry systems of Coatepec.

The Fisher's alpha and Shannon's index diversity analysis results differed in the present study. Both indexes consider the number of EF species (richness) and relative abundance of the individuals present in a given sample, but Fisher's alpha is not influenced by the sample size and is less affected by the abundance of the most common species than Shannon's index [82]. As such, Shannon's index indicated low variability among the four coffee plantations, whereas Fisher's alpha showed differences in diversity between the two regions and between agroforestry systems.

Although the total EF diversity found in the coffee plantations was not fully recovered, we found similar diversity values in other studies about EF diversity. In the present study, the mean of Fisher's alpha diversity of morphospecies (5.1–6.5) is similar to the mean of genotypic diversity (Fisher's alpha diversity of 5.2) of coffee plants in Mexico [16]. The similarity in diversity index values between the present study and other studies shows that morphotypes and morphospecies are valid taxonomic units [83] and that comparing the results of studies using different taxonomic units might be valid. The similitude in richness species estimators using morphotypes and genotypes has also been reported on foliar endophytes from beech trees (*Fagus sylvatica*) [79].

The Shannon's diversity values varied around 2 in the four coffee plantations. According to Gazis and Chaverri (2010) [84], the Shannon's index values in studies on EF are usually between 1.5 and 3.5; therefore, the Shannon's index for each coffee plantation in the present study is similar to those reported by other studies on EF.

In the analysis of association between species, the interactions between fungi are apparently different in each coffee plantation, but in general, the fungi isolated in high frequencies, such as *Colletotrichum gloeosporioides* 1 and *Xylaria* 1, have more negative co-occurrences than species isolated in low frequencies. The negative covariances between *Colletotrichum gloeosporioides* and *Xylaria* spp. have been reported previously for endophytes inhabiting coffee leaves [15].

Species found in high frequencies co-occur with a low number of species, whereas the species isolated in low frequencies co-occur with a high number of species. Our results are consistent with those of Pan and May (2009) [85], who reported higher negative species co-occurrences between dominant EF and higher positive covariances between less common fungal species in maize plants. The positive covariances between EF could be attributed to a lack of competitive exclusion between those fungi or to a phenomenon known as facilitation. Fungal facilitation in endophytic communities can occur when infection of a plant host by one fungus species makes that host more vulnerable to infection by another fungus species [85]. The interspecific interactions of EF could influence the diversity and assemblage of EFC found in each coffee plantation.

The NMDS plots based on the different ecological indexes showed variation in EFC among the different plants of each coffee plantation. In addition, there was no EFC clustering among coffee plantations. When the EFC similarities of the coffee plantations were compared with an ANOSIM test, we found that the geographically closest plantations, the ones in the Coatepec region, were similar in their EFC, independent of their agroforestry systems. In agreement with this result, some studies have shown that EFC similarity is a function of the distance between sites, and thus, nearby sites have similar EFC [27], [86], [87]. The geographical condition of this region (microclimate and surrounding vegetation) could influence the assemblage of the EFC.

In contrast, we found dissimilarities in the EFC of the two Huatusco plantations, even though they were near each other. The ANOSIM test showed that the EFC of RP-H and SP-H were different according to the three ecological indexes of similarity that were analyzed. The EFC of RP-H and SP-C were also different as analyzed using Euclidean distance. Those results suggest that agroforestry system could influence the assemblage of EFC of coffee plants. Our results also showed that the EFC of the rustic plantations and the EFC of the simple polycultures were similar; in fact, the rustic plantations shared a higher number of morphospecies than the simple polycultures did.

Studies on the EF of herbaceous cultivable plants, such as cotton and maize, have shown no similarities in EFC based on farming practice [38], [88]. However, in agreement with our results, Pancher et al. (2012) [37] reported dissimilarity in EFC isolated from *Vitis vinifera* plants under different vineyard management practices. These results indicate that the plantation management techniques in agroforestry systems, such as coffee plantations and vineyards, might influence the assemblage of EFC.

More sampling will help elucidate the factors that influence the EFC, but as shown in this study, some of those factors might be the geographical location and agroforestry system of the coffee plantations. Further studies evaluating the ecological roles of the different EFC found in the diverse coffee agroforestry systems will contribute to the knowledge of the role of the system of agroforestry management in the regulation of pest and pathogen populations [89], [90].

The results of the present study show that the coffee agroforestry system produces variability in the colonization, richness, diversity, and composition of EFC in coffee plants. In addition, they demonstrate that the region in which coffee is cultivated is an important factor that influences these parameters. Future studies on the biodiversity conservation value of different coffee agroforestry systems will need to consider the coffee region as a determinant factor that affects biodiversity. In further studies, the use of molecular and physiological tools to identify, individually and collectively, the functional and ecological significance of EF in coffee plants under diverse ecological and geographical conditions will be equally significant. These studies will also provide an opportunity to understand the potential use of some EF as producers of relevant precursor substances in the regulation of different pests and pathogens, to discover new drugs, and to understand the potential role of EFC as potential controls of pest populations.

## Supporting Information

Table S1 Description of morphospecies of foliar endophytic fungi isolated from the four coffee plantations.
(DOCX)
