# Supplementary material for: Diversity and Communities of Foliar Endophytic Fungi from Different Agroecosystems of Coffea arabica L. in Two Regions of Veracruz, Mexico
Source: PLoS One. 2014 Jun 2;9(6):e98454. doi: 10.1371/journal.pone.0098454 (PMC4041768; doi:10.1371/journal.pone.0098454)
Supplement: Table S1 — Description of morphospecies of foliar endophytic fungi isolated from the four coffee plantations. (DOCX) [file pone.0098454.s001.docx]

**Table S1.** **Description of morphospecies of foliar endophytic fungi isolated from the four coffee plantations**.

| Taxon | Description |
| --- | --- |
| *Alternaria* *citri* | Mycelium velvety, green olivaceous to black; conidiogenous cells integrated and cylindrical; conidia solitary and in simple branches, dry, subspherical to irregular, black, verrucose, muriform, (15-) 16-37 (-43) x (8.5-) 10.5-20 (-23.5) µm. |
| *Beauveria* *brongniartii* | Mycelium powdery, white; conidiogenous cells flask-shaped; the conidia are borne along a simpodial rachis of zigzag form; conidia hyaline, ovoid, (2.7-) 3-5 X (1.6-) 2-3 µm. |
| *Colletotrichum* aff *brassicicola* | Mycelium flat and hyaline, few aerial mycelia olive to black coloured; black conidiomata poorly developed, thin walled; conidiogenous cells enteroblastic, often extending to form new conidiogenous loci (indeterminated), cylindrical, 10-18 x 3-4 µm; conidial masses reddish yellow, conidia ellipsoid to oblong-ellipsoid, hyaline, apex obtuse and base truncate, (13-)14-16 (-17) x 4.5-5.5 (-5.8) µm. |
| *Colletotrichum gloeosporioides* 1 | Mycelium fluffy to lanose, white to pink-coloured; conidioma stromatic and black; setae present, pigmented, 47-78 x 2.7-3.8 µm; conidiogenous cells enteroblastic, cylindrical, 7.5-14.5 (-20) x 3-5 (-6) µm; conidial masses reddish yellow; conidia obovate to oval, with 1-2 guttules, straight to slightly curved, obtuse at the apices, with a hilum-like at the base, (10-) 12-15 (-17) x 4.5-6.5 (-8) µm; apresoria irregular, brown, 4-5.6 (-7.2) x 4.-5.6 (-7.2) µm. Positive to citrate and tartrate. |
| *Colletotrichum gloeosporioides* 2 | Mycelium irregular in morphology, flat and white to reddish yellow-coloured; conidioma stromatic and black; conidial masses reddish yellow; conidia produced in stromes and from fertile hyphae; conidiogenous cells enteroblastic, cylindrical, 20-22 x 3.2-4. 3 µm; conidia ovovate, straight to slightly curved, obtuse at the apices, with one guttula at the center, (12.6-) 15.61 (-19.8) x (4.9-) 5.9 (-7.5) µm. Positive to citrate and tartrate. |
| *Colletotrichum musae* | Mycelium fluffy, white to gray coloured; conidioma stromatic, thin walled; conidial masses light red; conidiogenuos cells enteroblastic, cylindrical, (11.4-) 14.98 (19.1) x (2-) 2.43 (-2.8) µm; conidia oblong-elliptic to cylindrical, hyaline, obtuse at the apices, straight to slightly curved, guttulated, (13-) 14.9 (-17.5) x (4.2) 4.83 (5.6) µm. Positive to citrate and tartrate. |
| *Colletotrichum* sp. | Mycelium fluffy to floccose, whitish gray to gray; conidioma stromatic, covered with fluffy gray mycelia, thin walled; conidiogenous cells enteroblastic, cylindrical; conidial drop reddish yellow, conidia hyaline, thin walled, ellipsoid, apex obtuse and base with a truncate scar, guttulated, 12-20 (-26) x 4-6 µm. Positive to citrate and tartrate. |
| *Coniosporium* sp. | Mycelium velutinate to floccose, greyish olivaceous; conidia formed by liberation of arthric cells, ellipsoidal, some slimming at the centre, truly muriform cells absent, 11.5-19 x 3.5-5.5 µm. |
| *Cryptopsoriopsis corticola* | Mycelium fluffy, gray to dark olivaceous; conidioma stromatic, thin walled, setae darkly pigmented, septated, 200-290 X 3-6 µm; conidiogenous cells enteroblastic, cylindrical to ampuliform; conidial masses pale rose, conidia oblong-ellipsoid, apex obtuse and base with a truncate scar, straight, hyaline, thin walled, guttulated, septated with age (2-6) and slightly curved, (28-) 31-45 (-48.4) x (8.5-) 9-11 (-12) µm. |
| *Cryptopsoriopsis* sp. | Mycelium lanose, gray olivaceous coloured; conidiomata stromatic, thin walled; conidiogenous cells enteroblastic, cylindrical to clavated, with a notable neck, indeterminated and some with two loci, (10.5-) 15-21 (-37) x (2-) 3-4.5 µm; conidial masses reddish yellow; conidia hyaline, thin walled, guttulated, ellipsoid to oblong-ellipsoid, apex obtuse and base with a truncate scar, (14.5-) 16-21 (-23) x 4.5-6 µm. |
| *Diplodia* sp. | Mycelium fluffy to floccose, olive gray to dark gray; black conidiomata, covered with fluffy gray mycelium; conidiogenous cells cylindrical to doliiform, enteroblasltic, 14-22 x (4.1-) 5.5-8 µm; conidia ovoid to oblong-ellipsoid, obtuse at the apices, straight, 17-22 (-26) x (6-) 7-9 µm; later becoming dark brown and septated, 3 to 4 septae, 20-25 (-41) x 7-9 µm. |
| *Glomerella* *cingulate* | Mycelium fluffy to floccose, white to whitish gray becoming olive gray with age; perithecia black; asci cilyndrical, (42-) 45-60 (-65) x 8-11 µm, with 8 ascospores; ascospores allantoid, with 2 guttules, (13.7-) 16-21 (-22) x 4-5 (-6.5) µm; black conidiomata; conidia oval, (8-) 10-15 (-17) x (3.2-) 4-5(-6) µm. |
| *Guignardia mangiferae* | Mycelium flat, olive-green to dark green coloured, margin smooth to undulate; stromata black and coralloid bearing pycnidia; asci bitunicate, clavate, 48.5-100 (-127) x 10-15.5 (-19.4) µm; ascospores obovate to rhombic, swollen in the middle, ends obtuse, each with a hyaline appendage, aseptate, guttulate, (9.5-) 10-17.3 (-18.3) x (4) 4.7-7.1 (-7.8) µm. Conidia obovate to elliptic, guttulate, hyaline, conidial appendages 8.1-10.8 (-14.7) x 6.4-7.6 µm. |
| Hyphomicete 1 | Mycelium flat, rose to red yellowish coloured; conidia arthric, hyaline, cylindrical with truncate ends, (8-) 10-45 x 2-3 µm. |
| Hyphomicete 2 | Mycelia cottony, white, zonate; porospores ovovate to oblong-elliptic, thin at the centre, septates (1-2 septae); (10-) 12-20 (-22) x 4-5.5 (-7.5) µm. |
| Mycelia esterilia 1 | Mycelium submerged, hyaline to white. Mycelium on OA and PDA covering Petri dish in more of 10 days. |
| Mycelia esterilia 2 | Mycelium lanose and white. Mycelium on OA and PDA covering Petri dish in more of 10 days. |
| Mycelia esterilia 3 | Mycelium submerged and hyaline, clamidospores present. Mycelium on OA and PDA covering Petri dish in more of 10 days. |
| Mycelia esterilia 3 | Mycelium cottony, white to pale rose; media rose to violet pigmented. Mycelium on OA and PDA covering Petri dish in more of 10 days. |
| Mycelia esterilia 4 | Mycelium pinkish white, butyraceous texture. Mycelium on OA and PDA covering Petri dish in more of 10 days. |
| Mycelia esterilia 5 | Mycelium immersed, yellow bright. Mycelium on OA and PDA covering Petri dish in more of 10 days. |
| *Paecilomyces* sp. | Mycelium pulvinate, white to pale yellow coloured; conidiophores with verticillate branchs, giving rise to 2-5 phialides; phialides cylindrical swelling at the base, (10-) 15-20 (-30) x (1.5-) 2-3 µm; conidia hyaline, ovoid to obovate, catenulated, (3.5-) 4 - 7 (-8) x 2-3.5 (-5) µm. |
| *Phomopsis arnoldiae* | Mycelium fluffy, pale green coloured; pycnidia aggregated, dark, thick walled; conidiogenous cells enteroblastic, cylindrical, determinated, hyaline; conidial masses pale rose; alfa conidia elliptical, hyaline, with 2 guttules, 5-7 x 1.3-2.3 (-2.6) µm; beta conidia filiform, hyaline, hamate, 17-26 x 1-2 µm. |
| *Phomopsis* sp. | Mycelium cottony to floccose, pale brown; pycnidia aggregated, thick walled, dark; conidiogenous cells enteroblastic, cylindrical, determinated, hyaline; conidial masses cinnamon; alfa conidia abundant, oval, hyalines, with 1-2 guttules, obtuse apex and basal conidiogenous scar, 5-7 (-7.5) x 1.7-2.5 (-2.7) µm; beta conidia hyaline filiform, hamate, (10-) 12-17.5 (-20) x (0.7-) 1-1.5 (-1.7) µm. |
| *Xylaria* 1. | Mycelium fluffy, white to whitish brown coloured; reverse pale rose to whitish brown; no production of stromes or conidiomes. |
| *Xylaria* 2. | Mycelium adpressed with plumy margin, zonate, white coloured, later becoming black carbonaceous; reverse alternating concentric zones of white and black, becoming black with age; not production of stromes or conidiomes. |
| *Xylaria* 3 | Mycelium white, fluffy to floccose; reverse yellow to pink coloured; sterile stromes cylindrical, white to pink. |
| *Xylaria* 4. | Mycelium adpressed with festooned margin, zonate, first white alternating with concentric black zones, black carbonaceous with age; reverse black; no stromes. |
| *Xylaria* 5. | Mycelium fluffy with plumy margin, first white, then with black carbonaceous zones; reverse white with black irregular spots; sterile stromes emerging from melanized mycelium, filiforms and black to dark grey. |
| *Xylaria* 6 | Mycelium fluffy, white with the centre becoming dark gray to grey coloured; reverse white with centre dark gray; sterile stromes, emerging from white mycelium, filiforms and white. |
